# A method for exposing rodents to resuspended particles using whole-body plethysmography

**DOI:** 10.1186/1743-8977-3-12

**Published:** 2006-08-15

**Authors:** Lindsay B Wichers, Allen D Ledbetter, John K McGee, Robert B Kellogg, William H Rowan, Julianne P Nolan, Daniel L Costa, William P Watkinson

**Affiliations:** 1 Department of Environmental Sciences and Engineering, School of Public Health, University of North Carolina, Chapel Hill, North Carolina 27599, USA; 2 Environmental Media Assessment Group, National Center for Environmental Assessment, Office of Research and Development, Research Triangle Park, North Carolina 27711, USA; 3 Pulmonary Toxicology Branch, Experimental Toxicology Division, National Health and Environmental Effects Research Laboratory, Office of Research and Development, US. Environmental Protection Agency, Research Triangle Park, North Carolina 27711, USA; 4 Alion Science and Technology Corporation, Research Triangle Park, North Carolina 27711, USA; 5 Office of Research and Development, U.S. Environmental Protection Agency, Research Triangle Park, North Carolina 27711, USA

## Abstract

**Background:**

Epidemiological studies have reported increased risks of cardiopulmonary-related hospitalization and death in association with exposure to elevated levels of particulate matter (PM) across a wide range of urban areas. In response to these findings, researchers have conducted animal inhalation exposures aimed at reproducing the observed toxicologic effects. However, it is technically difficult to quantitate the actual amount of PM delivered to the lung in such studies, and dose is frequently estimated using default respiration parameters. Consequently, the interpretation of PM-induced effects in rodents exposed via whole-body inhalation is often compromised by the inability to determine deposited dose. To address this problem, we have developed an exposure system that merges the generation of dry, aerosolized particles with whole-body plethysmography (WBP), thus permitting inhalation exposures in the unrestrained rat while simultaneously obtaining data on pulmonary function.

**Results:**

This system was validated using an oil combustion-derived particle (HP12) at three nominal concentrations (3, 12, and 13 mg/m^3^) for four consecutive exposure days (6 hr/day); a single 6-hour exposure to 13 mg/m^3 ^of HP12 was also conducted. These results demonstrated that the system was both reliable and consistent over these exposure protocols, achieving average concentrations that were within 10% of the targeted concentration. In-line filters located on the exhaust outlets of individual WBP chambers showed relative agreement in HP12 mass for each day and were not statistically different when compared to one another (p = 0.16). Temperatures and relative humidities were also similar between chambers during PM and air exposures. Finally, detailed composition analyses of both HP12 filter and bulk samples showed that grinding and aerosolization did not change particle chemistry.

**Conclusion:**

The results of this study demonstrate that it is possible to expose rodents to resuspended, dry PM via whole-body inhalation while these animals are maintained in WBP chambers. This new methodology should significantly improve the ability to assess dosimetry under minimally stressful exposure conditions.

## Background

It has been well established that a positive association exists between the levels of ambient particulate matter (PM) and the incidence of morbidity and mortality, particularly for those individuals with preexisting cardiopulmonary diseases [1-3]. Recent studies have further shown that individuals with cardiac conduction disorders and heart failure are also at increased risk for adverse myocardial events and death following exposure to elevated PM concentrations [4-7]. As a result of these studies, emphasis has been placed on elucidating biological modes of action to explain the observed effects, with careful attention paid to the link between PM and adverse cardiac events.

While a number of hypothesized pathways for PM-induced cardiovascular effects have been examined in both *in vivo *and *in vitro *systems, none have yet been substantiated. In numerous studies, limited diagnostic methods and relatively small numbers of animals in the exposure groups have necessitated the use of PM concentrations that are often considerably higher than those of atmospheric PM in order to amplify otherwise subtle physiological effects. Despite the scientific progress made in this area, the use of such overly high concentrations of PM as employed in these studies remains a major criticism [8]. It is also clear that particle exposure concentration is not a precise metric for inhaled dose. Substantial research efforts have been directed toward improving our scientific understanding of the deposition, translocation, and clearance of particles in the respiratory tracts of both humans and laboratory animals, such that concentration (and therefore dose) can be better linked to adverse health effects. To this end, a handful of studies [9-11] have been conducted using nose-only exposure systems that included individual chambers outfitted with pneumotachographs to determine aerosol deposition in rats.

However, the extra stress inherent in these studies due to the restraint imposed by the nose-only procedure likely impacts PM dose; and, furthermore, this exposure method does not easily extrapolate to the human condition. Lastly, in the attempt to more readily estimate inhaled dose, many researchers have begun to use dosimetric modeling software packages (such as the Multiple Path Particle Model; CIIT/RIVM) with default species-dependent respiration and deposition parameters. The capability to obtain actual respiratory values on individual *unrestrained *animals *during *exposure for use as input variables in dosimetric calculations would constitute a major improvement to the existing methods.

The primary objective of this study was to develop and validate a rodent inhalation system capable of exposing rats to dry, aerosolized particles while maintained in whole-body plethysmograph (WBP) chambers. This methodology, when combined with radiotelemetry procedures, would permit the simultaneous monitoring and acquisition of continuous cardiac and pulmonary physiological parameters in unanesthetized, unrestrained rats while exposed to PM. Given that the cardiovascular and pulmonary systems are highly interconnected, this interaction is likely to play an important role in discerning unstressed physiologically-based mechanisms responsible for adverse health effects following exposure to PM. The ability to simultaneously monitor cardiac and pulmonary functional parameters in *unrestrained *animals during exposure to PM should prove highly beneficial to the study of these effects.

**Figure 1:**
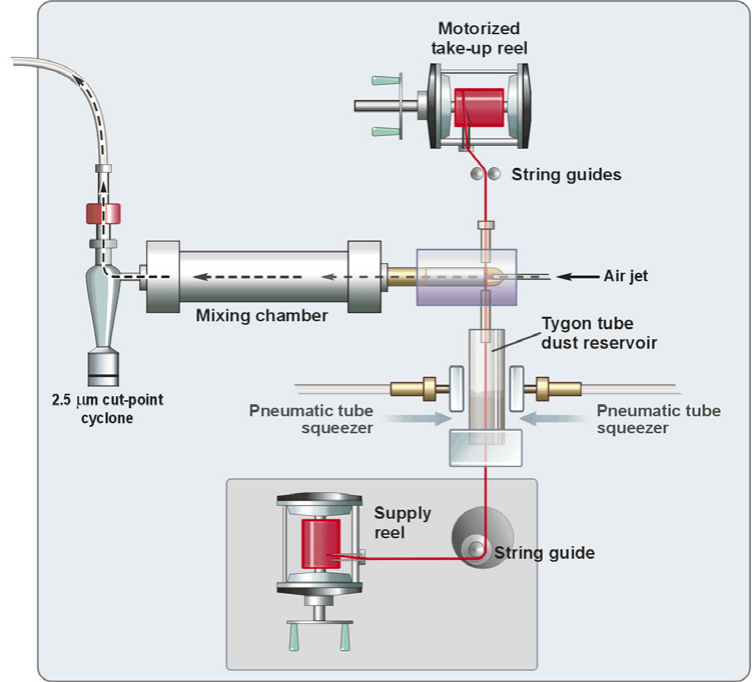
Schematic of string generator system (modified from Ledbetter *et al*. [12]).

**Figure 2:**
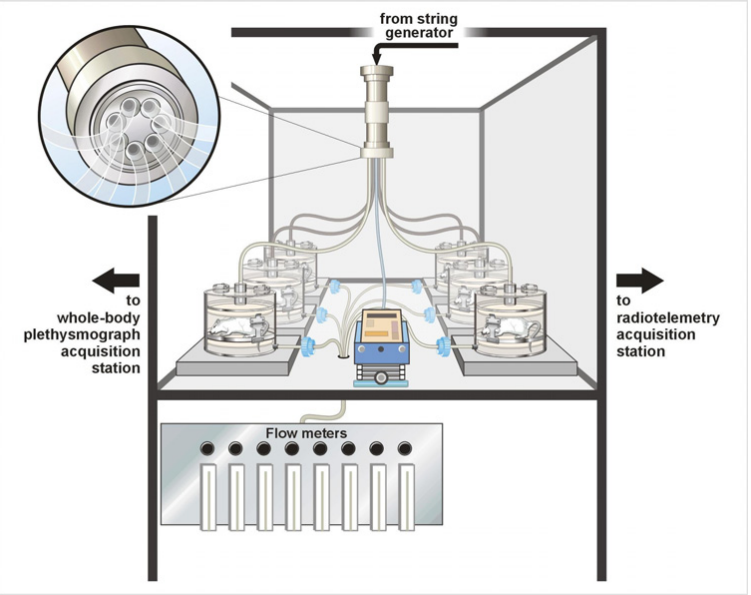
**Diagram of whole-body plethysmograph (WBP) particle exposure system**. Particles were resuspended using a string generator, then sent through a distribution head containing seven ports; six ports are connected to individual WBP chambers and the remaining port is used to assess particle concentration. The inset shows the distribution head design.

To accomplish this objective, we modified a dry particle string generator system (Figure 1) originally designed for nose-only inhalation exposures [12], to permit whole-body inhalation exposures within WBP chambers (Figure 2). The string generator has been shown to operate well under low air flow conditions and only requires a relatively small amount of material for aerosolization. The string generator also produces a stable and reproducible aerosol output [12]. The application of the string generator/WBP system to rodent inhalation studies permits more precise estimates of PM dose while also providing the ability to examine these effects using whole-body inhalation methods that better approximate the human scenario. These added capabilities should significantly improve the efforts to establish important linkages between PM exposure and adverse toxicologic outcomes.

Secondary objectives of this study were: 1) to fully characterize the composition of the model PM (HP12) in filter samples obtained from WBP chambers (initial bulk HP12 composition was previously reported [13]); and 2) to compare exposure filter sample findings to the bulk HP12 sample, in order to ascertain any changes in chemical composition due to aging, grinding, or aerosolizing. To our knowledge, only one other study has been conducted to evaluate the toxicity of PM components to the same extent as the current study, e.g., a recent study that examined samples obtained from the World Trade Center collapse [14]. Thus, the present study represents an important step in identifying PM characteristics that may provide further insight into toxic components, while also determining whether different chemical analyses yield consistent findings.

## Results and discussion

### Ambient conditions

Average temperatures were slightly higher in the one representative PM exposure WBP chamber compared to those of the control chamber (24.1°C vs 23.3°C, respectively) for all phases (Table 1). The maximal temperature range for the air and PM designated chamber across all phases was <4°C. Average 4-day relative humidity for the PM exposure chamber ranged from 10–13%. There was more variability in the control chamber, where observed relative humidity values were between 10 and 19%. The single greatest relative humidity value was observed for the control chamber during Phase 1 of our study (21%). This was likely (at least partially) attributable to an elevated breathing frequency for one control rat.

**Table 1:** Temperature and relative humidity for representative air and HP12 chambers averaged over the exposure day(s)

	**PHASE 1**	**PHASE 2**	**PHASE 3**	**PHASE 4**
Control Chamber Temperature (°C)	23.0	23.2	23.6	23.7
Control Chamber Relative Humidity (%)	20.6	9.8	16.2	14.3
Exposure Chamber Temperature (°C)	24.6	24.9	23.7	23.3
Exposure Chamber Relative Humidity (%)	12.7	12.0	9.7	7.0

The observed daily humidities of the string generator/WBP system were 7–13% in the representative PM exposure chamber and 10–21% in the control chamber. While these percentages were well below the recommended 50% for the housing of rodents, this discrepancy was purposeful. At higher humidity, the particles would be more likely to agglomerate, resulting in uneven distribution between chambers and exposure concentrations differing from target concentrations. As this exposure protocol was only carried out for 6 hr/day for four days, the rats spent the majority (75%) of each day in an approved animal facility where the humidity was maintained at ≈50%. The results of a previously-conducted subchronic nose-only inhalation study [15] examining this problem found no evidence of humidity-related effects when rats were exposed to 3% humidity intermittently for four weeks. Furthermore, in the present study, gross assessments of animal health (e.g., body weight and overall appearance, including examination for ringtail) were conducted daily both pre- and post-exposure to ensure that the low humidity was not having explicit detrimental effects. Thus, while the cumulative effects of low humidity may be of greater concern in a chronic study, the impact of low humidity on rat health in the present study was likely minimal.

### Chamber particle concentrations

Three 4-day exposures of six hours each were successfully completed at HP12 concentrations of 3, 12, and 13 mg/m^3 ^(Phase 1, 2, and 3, respectively). Additionally, a single 6-hr exposure was conducted at a concentration of 13 mg/m^3 ^(Phase 4). It should be noted that the original target HP12 concentration for the high exposure was 12 mg/m^3^, but as rats from Phases 3 and 4 were to be used to assess particle dose, the exposure concentrations for these phases needed to be the same. As 13 mg/m^3 ^was achieved for the 1-day exposure, that concentration had to be duplicated for the 4-day exposure.

Masses of HP12 collected on filters from the seventh port on the distribution head via gravimetric methodology provided the exposure concentrations (Figure 2). The low-concentration 4-day exposure (Phase 1) resulted in an actual average concentration of 3.3 ± 0.35 mg/m^3 ^over the entire exposure period, while the second 4-day exposure (Phase 2) resulted in an actual average concentration of 11.3 ± 0.63 mg/m^3 ^over the exposure period (Table 2). The average particle concentration over the four days of exposure during Phase 3 was 13.2 ± 0.06 mg/m^3^; for Phase 4, the average HP12 concentration for the one-day exposure was 13.1 mg/m^3^.

**Table 2:** Average daily HP12 concentrations (mg/m^3^) calculated from filter samples

	**PHASE 1**	**PHASE 2**	**PHASE 3**	**PHASE 4**
Day 0	3.7	9.6	13.2	13.1
Day 1	2.7	11.0	13.0	N/A
Day 2	2.9	12.3	13.3	N/A
Day 3	4.2	12.3	13.1	N/A
*Average*	*3.3*	*11.3*	*13.2*	*13.1*
RSD^a^	20.8%	11.1%	0.98%	N/A

^a^RSD = relative standard deviation

Filters were collected once every hour using gravimetric methodology during a 6-hr inhalation exposure for both 1- and 4-day study durations.

Since it was crucial to determine the particle concentration within the WBP chambers, and direct measurement of particle concentration was not possible, it was important to have both a primary and a secondary means of assessing this value. In this study, gravimetric filter data provided an accurate representation of HP12 exposure concentration. HP12 samples (accumulated over 5-min periods on filters) were collected hourly from a designated port of the vertical mixing chamber during exposure and appropriate adjustments were made to the string generator to maintain the target concentration. A real-time aerosol monitor was employed as a secondary method of estimating particle concentration during those times when a filter sample was not being collected, although these data were only used for making minor adjustments to the string generator. Thus, the accuracy of the estimates of chamber particle concentration were dependent solely upon the precision of the gravimetric filter measurements.

### Chamber particle distribution

**Figure 3:**
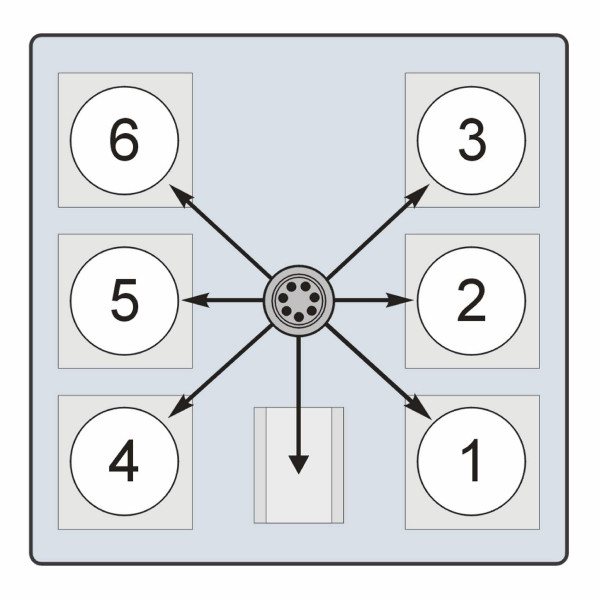
Overhead view of whole-body plethysmograph chamber configuration.

The average particle mass collected on filters for each WBP chamber is shown in Table 3 (configuration shown in Figure 3). There was good agreement for particle mass accumulated across all chambers, with an overall average of 0.744 ± 0.056 mg for Phase 1; all particle masses were within 27% of the average. In general, the average daily accumulated mass increased throughout the exposure duration for Phase 1; however, this was not the case for Phases 2 or 3. For Phase 2, the average mass of PM collected for all chambers and exposure days was 1.657 ± 0.075 mg. There was much better agreement between exposure chambers for Phase 2, as all collected masses were within 6% of the overall average. Phase 3 had relatively high deposited PM mass on individual days for Chambers 2 (4.739 mg on Day 3) and 3 (5.425 mg on Day 0), when compared with other chambers. While there were some elevations in accumulated PM mass observed on isolated days in specific chambers (i.e., 5.425 mg in Chamber 3 on Day 0 of Phase 3), this finding was not consistent across exposure days, phases, or chambers. Consequently, when tested statistically, there were no significant differences in mass accumulation between exposure chambers. Overall, a positive relationship between exposure concentration and filter mass was observed, such that the average accumulated HP12 mass in Phase 3 was three times that of Phase 1.

**Table 3:** Daily and average HP12 mass (mg) collected on gravimetric filters

	Chamber 1	Chamber 2	Chamber 3	Chamber 4	Chamber 5	Chamber 6	Average	RSD^a^
**Phase 1**								
Day 0	0.620	0.486	1.022	0.526	0.310	0.551	0.586	40.6%
Day 1	0.867	0.656	0.848	0.617	0.575	0.447	0.668	24.3%
Day 2	0.973	0.634	0.656	0.765	0.860	0.763	0.775	16.4%
Day 3	1.497	0.686	0.919	0.856	1.158	1.285	1.067	28.2%
Average	0.989	0.616	0.861	0.691	0.726	0.761	**0.774**	--
RSD	37.3%	14.4%	17.9%	21.4%	50.3%	49.0%	--	**35.5%**
**Phase 2**								
Day 0	2.473	1.201	1.701	1.427	1.568	1.456	1.638	27.0%
Day 1	1.706	1.671	1.601	2.111	1.986	1.175	1.708	19.2%
Day 2	0.926	1.513	1.902	1.627	1.849	--^b^	1.563	25.0%
Day 3	1.496	2.062	1.832	1.529	1.244	2.135	1.716	20.4%
Average	1.650	1.612	1.759	1.674	1.662	1.589	**1.657**	--
RSD	38.8%	22.2%	7.63%	18.1%	19.8%	31.1%	--	**21.6%**
**Phase 3**								
Day 0	1.214	2.677	5.425	2.971	2.283	2.634	2.867	48.6%
Day 1	2.715	1.973	4.406	2.997	1.141	2.740	2.662	41.0%
Day 2	1.287	1.902	3.038	1.941	1.152	2.287	1.935	35.6%
Day 3	1.457	4.739	1.602	2.050	2.279	1.510	2.273	55.1%
Average	1.668	2.823	3.618	2.490	1.714	2.293	**2.434**	--
RSD	42.3%	46.9%	45.9%	23.0%	38.2%	24.3%	--	**46.1%**
**Phase 4**								
Day 0	1.780	1.783	2.661	3.038	1.762	3.673	2.450	32.9%

^a^RSD = relative standard deviation

^b^Particle mass was not obtained for this day.

Filters were attached in-line to the exhaust of individual whole-body plethysmograph chambers (see Figure 2). Accumulated mass was obtained for single 6-hr exposures.

It was not possible to sample flow rates in the individual exposure chambers (and thus obtain precise calculations of HP12 concentrations of WBP chambers) during exposure and as noted above, based on the HP12 mass collected on filters, there were no significant differences between chambers. However, the data indicate there were some slight variations in PM mass that could be attributable to a number of sources including animal positioning relative to the particle distribution port located on the top of individual WBP chambers or random particle surges from the vertical mixing chamber when target concentrations were momentarily exceeded. Although the Tygon™ tubing from the distribution chamber to each WBP was of equal length and diameter, particles may have deposited on the tubing walls in an uneven manner that was possibly due to bends in the tubing. Finally, there could also have been incomplete mixing within the distribution chamber, resulting in variable PM concentration at the distribution head.

### Pulmonary function

In general, breathing frequency (*f*), tidal volume (V_T_), and minute ventilation (MV) decreased slightly over the four days of exposure for all groups throughout each Phase. Average *f *values over the 6-hr HP12 exposure ranged from 66 to 103 breaths/min, V_T _was 1.1 to 2.4 ml, and MV was 97 to 190 ml/min. Despite the individual rat variability, the overall mean pulmonary function parameters were similar across Phases, with *f*, V_T_, and MV approximately 90 breaths/min, 1.5 ml, and 130 ml/min, respectively. The wide ranges of observed values were likely attributable to differences in age, as rats were 11–17 weeks old. Although statistically significant decreases (≈15 breaths/min on the last two days of exposure) were observed in *f *for rats exposed to 12 mg/m^3 ^HP12, it appeared that this was an adaptation response and that lung function was not impaired, as there were no changes in other WBP parameters.

### Particle composition

**Figure 4:**
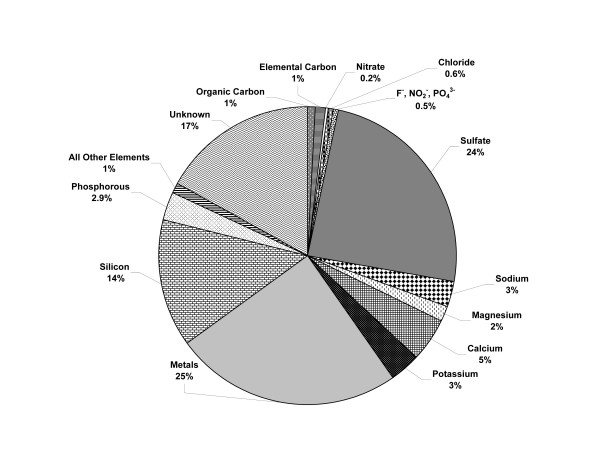
**Chemical composition of HP12 by percent of total mass**. Determination of constituents was conducted using X-ray fluorescence, carbon fractionation, inductively-coupled plasma optical emission spectrometry, and ion chromatography. The metals category includes Al, Cu, Fe, Mg, Ni, V, and Zn. The unknown portion is likely comprised of moisture, along with oxygen and hydrogen associated with organic and inorganic fractions [14].

Bulk HP12 is similar to other residual oil fly ashes in that it has relatively high levels of transition and heavy metals compared to ambient PM (Figure 4). However, few of these metals show appreciable water-solubility (Al, Cr, Fe, Co, Ni, and Zn), while the remainder are largely insoluble in water, but are soluble in nitric acid (V, Cu, Mo). Those elements found to be water soluble in HP12 are considered "easily bioavailable" and those soluble in HCl are classified as "totally bioavailable." The combined analytical methods of X-ray fluorescence (XRF), carbon fractionation (CF), inductively-coupled plasma optical emission spectrometry (ICP-OES), and ion chromatography (IC) resulted in a thorough characterization of both bulk and resuspended HP12 (with a total of 62 analytes measured) and accounted for 83% of HP12 mass. Of this, ≈2% was organic and elemental C. The remaining unknown mass fraction (17%) was likely attributable to: 1) moisture content; and 2) oxygen and hydrogen associated with organic and inorganic fractions [14].

**Figure 5:**
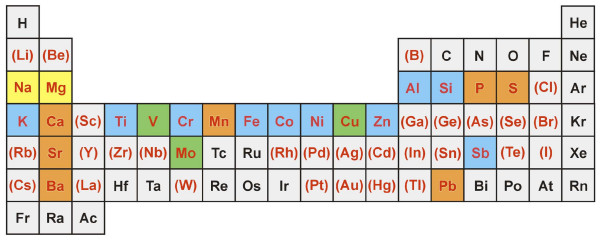
**Periodic table outlining chemical analyses of HP12**. Those elements shown in red were analyzed using X-ray fluorescence (XRF) and/or inductively-coupled plasma optical emission spectrometry (ICP-OES). Elements in red with parentheses (i.e., (B)) are indicative of elements near or below instrument detection limits. Elements shaded in: 1) yellow represents those that are completely soluble elements (based on deionized water and 1 M HCl extracts using ICP-OES); 2) orange represents completely soluble elements (based on 1 M HCl extract and XRF); 3) green represents those with good agreement between ICP-OES (1 M HNO3 extracts) and XRF; and 4) blue represents those found to be only partially soluble based on ICP-OES extractions.

Analyses using XRF and/or ICP-OES methodologies provided the capability to identify a total of 52 elements (Si measured as SiO_2 _and S measured as SO_4_) in the HP12 sample (Figure 5; Ce not shown). Of these 52 elements, 20 were present in sufficient concentrations to compare soluble levels to total content. As expected, there was general agreement between XRF and ICP-OES (Figure 5, Table 4); seven elements were completely soluble by deionized water or HCl extraction (P, S, Ca, Mn, Sr, Ba, and Pb), although the water-extract contents of P, Mn, Sr, Ba, and Pb were at least 15% lower than those obtained using XRF (albeit the amounts of these elements in HP12 were relatively small). Three elements were completely soluble based on nitric acid extraction (V, Cu, Mo); the stronger acid digest was done for comparative purposes with XRF results. The remaining ten elements (Al, Si, K, Ti, Cr, Fe, Co, Ni, Zn, and Sb) had XRF values greater than the highest ICP-OES extract values, indicating that these elements were only partially soluble in water. Of these partially soluble elements, Al, Si, Ti, and (to a lesser extent) Fe tended to form mixed-element, refractory oxides which do not easily go into solution. The metalloid Sb behaved in a similar fashion, and was largely insoluble in all extractions. The remaining transition metals were one-third to one-half soluble during extractions. Additionally, Mg and Na were completely soluble, based on water and HCl extractions; these elements were not analyzed by XRF, as their atomic numbers are too low for detection using this method.

**Table 4:** Comparison between different chemical analysis techniques and extractions using HP12 collected on filters

	**ICP-OES**			**XRF**
**Analyte**	**Deionized Water (n = 7)**	**1 M HCl (n = 7)**	**1.3 M HNO_3 _(n = 5)**	**Total Content (n = 5)**

**Al**	3.34(30%)	15.8(4.9%)	20.4(1.6%)	47.4(12%)
**B**	0.031(27%)	0.053(15%)	0.094(10%)	--*
**Ba**	2.01(4.9%)	**2.31(4.1%)**	**2.35(2.2%)**	**2.52(14%)**
**Ca**	**38.8(4.6%)**	**42.8(5.8%)**	**41.3(2.9%)**	**46.1(7.7%)**
**Co**	0.348(4.5%)	0.742(4.2%)	1.02(3.5%)	1.59(12%)
**Cr**	0.051(190%)	0.124(11%)	0.236(11%)	0.494(23%)
**Cu**	0.501(12%)	1.31(8.8%)	**2.22(6.4%)**	**2.49(8.9%)**
**Fe**	1.09(23%)	14.8(5.3%)	24.7(5.0%)	47.0(9.1%)
**K**	2.14(6.2%)	1.97(8.0%)	2.34(3.2%)	3.30(7.0%)
**Mg**	**13.9(4.8%)**	**17.0(6.0%)**	**16.4(2.6%)**	--*
**Mn**	0.315(3.2%)	0.442(4.4%)	**0.527(2.0%)**	**0.559(20%)**
**Mo**	0.473(7.4%)	1.64(4.0%)	**2.35(2.0%)**	**2.76(12%)**
**Na**	**31.5(7.5%)**	**27.6(6.9%)**	**30.3(8.0%)**	--*
**Ni**	6.61(5.4%)	15.7(4.5%)	28.2(3.3%)	43.1(8.9%)
**P**	2.06(14%)	**21.2(4.5%)**	**22.6(2.3%)**	**28.9(6.2%)**
**Pb**	0.162(14%)	**2.42(4.7%)**	**2.21(3.0%)**	**2.76(7.5%)**
**SO_4_**	**245(4.2%)**	**259(4.7%)**	**214(3.5%)**	**259(6.9%)**
**Sb**	0.008(270%)	0.190(5.2%)	0.287(8.1%)	1.81(14%)
**SiO_2_**	2.02(54%)	5.10(13%)	15.3(7.3%)	139(3.1%)
**Sr**	0.378(4.8%)	0.410(4.2%)	**0.412(3.1%)**	**0.455(20%)**
**Ti**	0.008(79%)	0.347(4.3%)	0.645(3.6%)	1.78(22%)
**V**	7.18(8.7%)	33.7(4.2%)	**54.6(3.2%)**	**62.9(7.3%)**
**Zn**	11.5(3.8%)	18.2(6.8%)	18.8(2.5%)	29.9(8.0%)

* indicates those elements with atomic numbers below which can not be assessed using XRF

All values are above instrument detection limits. Data are presented as means (RSD) and have units of μg/mg. Boxes with bold text indicate agreement within 15% between analyses methods for that element.

The strongly electropositive, ionic, highly reactive alkali elements are expected to be soluble in aqueous solvents [16,17]. P and S commonly form water-soluble, oxidized compounds or convert to these compounds in solution, particularly acidic solutions. In atmospheric PM, S is usually in the form of sulfate [18,19], and was found to be completely soluble in all three extraction liquids. The observed equivalent sulfate levels obtained by all extraction treatments and both instrumental methods provided an additional measure of quality control for analytical chemistry in this study.

The concentrations of the different elements in HP12, as determined by XRF and ICP-OES using deionized water and acid solutions of the PM, were consistent across all WBP exposure chambers, particularly for the transition metals commonly found in oil fly ashes (Fe, Ni, V, and Zn). Relative standard deviations (RSD = SD/mean × 100), an indicator of precision, ranged from 2.5% for Zn in 1.3 M HNO_3 _extracts to 23% for Fe obtained in deionized water analyzed by ICP-OES. Using XRF, the RSD of HP12 elemental content from different WBP chamber filters was <10% for Fe, Ni, V, and Zn (9.1, 8.9, 7.3, and 8.0%, respectively); RSD for the 1 M HCl-soluble extractions using ICP-OES were between 4.2 and 6.8% (V and Zn, respectively).

The combination of analyses determining total elemental content (e.g., XRF) with those targeting soluble elemental content (e.g., ICP-OES) provides a more detailed picture of HP12 composition which may be used to better estimate elemental speciation, potential bioavailability, and particle retention during inhalation. Water-soluble elemental content is associated with ready bioavailability and acute toxicity, whereas weak acid-soluble elemental content is associated with the absolute, long-term bioavailability [20,21]. The remaining elemental content, calculated by subtracting acid-soluble content from total elemental content, is considered inert and unavailable to the host.

There were no major differences in the bulk and filter HP12 elemental composition for 1 M HCl and 1.3 M HNO_3 _extracts (Table 5), indicating little elemental enrichment or depletion during generation of the HP12 aerosol. For toxicological purposes, this represents an important finding that facilitates comparison of the results of this inhalation study with those of previous intratracheal instillation studies using bulk HP12 [13,22,23].

**Table 5:** Comparison between bulk (n = 4) and filter (n = 7) HP12 samples using ICP-OES, 1 M HCl and 1.3 M HNO_3 _extractions

**ANALYTE**	**1 M HCl**		**1 M HNO_3_**	
	
	**FILTER (μg/mg)**	**BULK (μg/mg)**	**FILTER (μg/mg)**	**BULK (μg/mg)**
Al	15.8(4.9%)	17.2(1.0%)	20.4(1.6%)	20.9(1.4%)
B	0.053(15%)	0.120(6.0%)	0.095(10%)	0.144(7.2%)
Ba	2.31(4.1%)	0.762(0.9%)	2.35(2.2%)	1.52(1.0%)
Ca	42.8(5.8%)	40.6(1.0%)	41.3(2.9%)	41.1(1.3%)
Co	0.742(4.2%)	0.638(1.9%)	1.02(3.5%)	1.03(1.0%)
Cr	0.124(11%)	0.110(16%)	0.236(11%)	0.133(2.8%)
Cu	1.31(8.8%)	0.998(0.8%)	2.22(6.4%)	2.22(1.9%)
Fe	14.8(5.3%)	13.6(0.3%)	24.7(5.0%)	22.8(1.2%)
K	1.97(8.0%)	1.81(0.7%)	2.34(3.2%)	1.97(1.4%)
Mg	17.0(6.0%)	16.4(0.5%)	16.4(2.6%)	17.4(1.7%)
Mn	0.442(4.4%)	0.438(0.5%)	0.526(2.0%)	0.518(1.4%)
Mo	1.64(4.0%)	1.49(0.7%)	2.35(2.5%)	2.23(2.0%)
Na	27.6(6.9%)	20.5(2.8%)	30.3(8.0%)	19.5(2.3%)
Ni	15.7(4.5%)	13.1(0.5%)	28.2(3.3%)	27.6(1.1%)
P	21.2(4.5%)	21.1(0.2%)	22.6(2.3%)	23.0(2.7%)
Pb	2.42(4.7%)	2.06(0.8%)	2.21(3.0%)	2.31(1.7%)
SO_4_	259(4.7%)	222(0.8%)	214(2.5%)	218(2.8%)
SiO_2_	5.10(13%)	9.68(2.2%)	15.3(7.3%)	14.4(1.3%)
Sr	0.410(4.2%)	0.383(0.4%)	0.412(3.1%)	0.404(0.3%)
Ti	0.347(4.3%)	0.370(0.9%)	0.645(3.6%)	0.726(1.4%)
V	33.7(4.2%)	31.4(0.5%)	54.6(3.2%)	52.7(1.3%)
Zn	18.2(6.8%)	15.5(0.6%)	18.8(2.5%)	19.0(1.2%)

All values are above instrument detection limits; data are not shown for As, Be, Cd, Li, Sb, Se, Sn, and Tl as concentrations for these elements were below detection limits. Data are presented as means (RSD), where RSD = relative standard deviation.

The toxicities of oil fly ashes such as HP12 have largely been ascribed to relatively high levels of soluble transition metals, such as V, Fe, Ni, and Zn [20,21,24]. Previous toxicology studies employing ROFA have often been criticized for their use of non-atmospheric particles; however, unlike studies conducted using concentrated ambient particulates, which can have wide daily fluctuations in PM composition and concentration, research involving a well-characterized, surrogate PM is useful in advancing the understanding of the possible mechanisms associated with PM-induced health effects. Thus, the more comprehensive the chemical analysis of a given PM, such as HP12, the more useful it will be in future laboratory studies as an appropriate surrogate for ambient PM. As discussed in previous publications from our laboratory [13,23], HP12 has a composition profile more similar to that of ambient PM than to the ROFA used previously in many laboratories, primarily due to the smaller quantities of water-soluble metals (including Ni, V, and Zn). This is supported by comparisons with aerosol composition data collected at 13 U.S. speciation sites from 2001–2002 which demonstrated that the transition metals, Al, Fe, Mg, Si, and Zn, were found in the greatest quantities at these locations [25]. Admittedly, the concentrations of these metals in HP12, as measured by XRF, are much higher than those in the ambient PM taken from the speciation monitoring sites; however, as it has been shown that environmental PM contains water-soluble metals, a useful comparison may be made between the two particle types.

There are slight differences in the chemical composition of the bulk HP12 described in Wichers *et al*. [13] and that in the present study, largely due to the more comprehensive analyses done for the current research. In our previous work, ICP-OES was conducted on deionized water and 1 M HCl extracts to analyze for S, Al, Ca, Cu, Fe, Pb, Mg, Ni, K, Na, V, and Zn only; thus, the addition of XRF methodology for determining particle content resulted in >83% characterized HP12 mass (compared to 44% previously). The levels of sulfur (as sulfate) were similar in the prior and current analyses (22 and 24%, respectively), as were the amounts of C (2.1 and 1.9%, respectively), Ca (3.9 and 4.6%, respectively), and Na (2.5 and 2.8%, respectively). The previously determined total metal concentrations (Al, Cu, Fe, Mg, Ni, Pb, V, and Zn) in HP12 were 3.3 and 12% for water- and HCl-leachable solutions, respectively (although Pb and Fe were below detection limits for deionized water extracts); the same analysis conducted in the present work resulted in 5.1% and 14% total metal contents for water and HCl extracts, respectively. However, when XRF data are considered, the percent mass accounted for by these metals increases to 25%. Based on the XRF analysis, the remaining "unknown" fraction of HP12 mass (as classified in Wichers *et al*. [13]) consisted of Si (14%), P (2.9%), and 11 other elements (1.2%).

## Conclusion

The successful development of an inhalation system which incorporates WBP chambers, such that noninvasive pulmonary function parameters can be obtained from rodents while being exposed to resuspended, dry particles, provides a substantial advancement in PM exposure methodologies. While this study is not the first to combine an aerosol generation system with plethysmography, to our knowledge, all of the other exposure designs utilized nose-only inhalation methodology. It has been reported that the restraint inherent in nose-only systems induces significant increases in heart rate and core temperature in rats and mice [26]; thus, it is feasible that restraint-associated stress could alter ventilatory parameters, a major drawback when these parameters are subsequently used in evaluating deposition. Not only does the current system permit the collection of more physiologically relevant ventilatory parameters necessary for the estimation of particle deposition *during *whole-body inhalation exposure, it also provides the capability to acquire cardiovascular physiology data via radiotelemetry, resulting in dose-to-effect linkages with these parameter values.

Calculation of the delivered dose (D) of inhaled particles (D = C × d_f _× t × *f *× V_T_) to the thoracic and pulmonary regions can be estimated given chamber concentration (C), deposition fraction (d_f_), duration of exposure (t), breathing frequency (*f*), and tidal volume (V_T_). In most animal inhalation studies using PM, rats are group housed in large exposure chambers engineered for even particle distribution and optimal system performance. The use of multiple animals in large chambers precludes the collection of ventilatory data, and therefore delivered dose, in individual rats during exposure (although measures of pulmonary function are sometimes collected pre- and post-exposure to provide informed estimates of dose). The development of the capability of whole-body inhalation in the small WBP chamber adds an important component to PM dosimetry, as it permits the use of actual respiration data in predictive models to establish a better linkage between PM exposure, dose, and toxicological effects. Additionally, if the specific dose to the pulmonary region can be derived from airborne PM concentration, the uncertainty in extrapolating from adverse effects observed in laboratory rodents to humans will be considerably diminished. With this exposure system, it is also possible to ascertain if there are alterations in pulmonary function during exposure, if these changes are concentration-dependent, and how they might affect dose.

The measured PM concentrations in the above replicates demonstrate that the string generator exposure system is both reliable and consistent. When these data are compared to earlier string generator concentration and particle data presented by Ledbetter *et al*. [12], the RSD of the particle concentrations of this study (20.8 and 11.1% for Phase 1 and Phase 2, respectively) are within the same range as those reported previously (7.5–28.3%). The RSD for Phase 3 was much lower than these values, e.g., 0.98%, which may be attributable to the decreased variability in concentration across exposure days that was observed with increasing target concentration.

Based on the results of this study, there are a number of advantages gained by pairing a string generator with a WBP system for rodent PM exposures (Table 6). Our data show that the aerosol generation system performs well and is capable of maintaining consistent concentrations over a range of values suitable for rodent PM inhalation exposures. While there are physical/practical limitations with respect to the minimum (1.3 mg/m^3^) and maximum (33 mg/m^3^) concentrations that can be attained using the string generator [12], the achieved concentrations for this study were well within these ranges. As the WBP system was not originally designed for dry particle exposure, the creation of a reliable aerosol exposure system that readily connects to the WBP chambers provides a significant enhancement to the original WBP capabilities.

**Table 6:** Benefits and limitations of the WBP exposure system

**BENEFITS**	**LIMITATIONS**
• whole-body inhalation • real-time acquisition of respiratory data during exposure • capability to generate mixtures, as well as particles • simultaneous collection of pulmonary and cardiovascular data • can be modified for mice or rats	• low humidity • not able to obtain air flow data for individual WBP chambers during exposure • differences in particle distribution across WBP chambers • not able to expose large groups of animals simultaneously • equipment and space requirements • particles used must be from a bulk sample

Due to the constraints of our WBP system, we were only able to use eight chambers at a time. Thus, our current exposure capabilities require that studies be conducted in replicates such that six rats are exposed to PM while two control rats receive air. However, it may be possible in the future to increase the number of chambers to the maximum hardware capacity of 32. The system could also be easily modified to accommodate mice exposures by incorporating smaller WBP chambers. Of course, other considerations must be made when expanding or altering the system, such as particle generation and distribution, air flow, and sampling requirements, and space/equipment needs.

Of late, growing emphasis has been placed on refocusing PM research on "real-life" atmospheric exposure conditions, and particularly on using a multi-pollutant approach [8,27]. This PM exposure system was designed with the capability of exposing animals to particles plus gases and, thus, this system could easily incorporate additional air pollutants (i.e., criteria and/or hazardous air pollutants) for investigating atmospheric "mixtures". Depending upon gas properties, different requirements may be necessary for preparing the exposure system, which could include saturating the chambers for reactive gases (i.e., ozone) or coating the inside of exposure chambers to prevent adsorption. Therefore, the primary limitation on multi-pollutant exposures using this system is likely to be reactivity with the material from which the WBP chambers are manufactured.

In summary, the results of this work demonstrate that the particle generation system developed in our laboratory for individual whole-body inhalation exposures in rodents performs well over a range of concentrations suitable for rodent PM studies. These results further show that particle distribution (as measured by accumulated HP12 mass) across individual exposure chambers is remarkably similar. Finally, the more complete chemical characterization of HP12 promotes the elucidation of the possible linkages between adverse effects of PM and harmful constituents, and should provide valuable information as this particle is employed in future animal toxicological research.

## Methods

### Animals

Male, Spontaneously Hypertensive (SH) rats were obtained from Charles River Laboratories and ranged in age from 11–17 weeks at the start of each phase. Animals were housed singly (if the animal had been implanted with a radiotelemeter) or in pairs (if not implanted with a radiotelemeter) in plastic cages with beta-chip bedding. The relative humidity was maintained at 50 ± 5% and the ambient temperature at 22 ± 1°C, in accordance with standards established by the Association for Assessment and Accreditation of Laboratory Animal Care. A 12-hr light:12-hr dark cycle (0600-1800:1800-0600) was also imposed for all phases. Rats received laboratory feed (Purina rat chow) and water *ad libitum *from time of receipt to time of sacrifice. Animals did not have access to food or water while maintained in the WBP chambers.

### Experimental protocol

Eight WBP chambers (Model PLY3213; Buxco Electronics, Inc.; Sharon, CT) were used per phase (two air and six exposure) and each chamber was calibrated daily prior to animal loading. Rats were exposed for 6 hr/day (0800-1400) with the first day serving as a control day (Day -1), during which all animals received filtered room air. Days 0–3 were exposure days for animals designated for treatment; control animals received filtered air only. At the cessation of each daily exposure, animals were taken out of the chambers and returned to their home cages. The chambers were then washed, dried, and sprayed with a non-static spray solution (Staticide^®^; ACL; Elk Grove Village, IL) in preparation for the next day.

### Aerosol generation methodology

A string generator system [12] was used to resuspend dry HP12 for distribution to each WBP chamber (Figures 1 and 2). The generator operates by pulling a cotton string through a particle-filled reservoir, allowing particles to adhere to the string. The particle-laden string proceeds through a discharge head where compressed air blows particles off the string into a horizontal mixing chamber, then into a cyclone, a vertical mixing/distribution chamber, and finally into the WBP chambers.

The string (South Maid, 100% Mercerised Cotton, Size 10; Coates & Clark; Greenville, NC) for the generator was stored on a fishing reel (Penn Model 109 M; Penn Fishing Tackle Mfg.; Philadelphia, PA) inside a dry air-bathed container (Figure 1). The string passed through a Tygon™ tube (length = 2.5 inches; inner diameter = 0.375 inches) that served as the particulate reservoir, through a particulate discharge chamber, and accumulated on the take-up reel. The movement of the take-up reel was controlled by a stepper motor that pulled the string in incremental steps, resulting in the slow advancement of the string through the system; the exposure concentration was controlled by adjusting the speed of the stepper. As the string passed through the dust reservoir, two opposing squeezers slightly compressed the reservoir walls to ensure maximum contact between particulate and string and to prevent "channeling" within the reservoir. Inside the discharge head, an air jet blew the PM off the string into a horizontal cylindrical mixing chamber and through a 2.5 μm cut-point cyclone (Model URG-2000-30-EN; University Research Glassware; Chapel Hill, NC). The respirable PM then entered a vertical cylindrical mixing chamber (Figure 2) outfitted with a distribution head (designed and fabricated in-house) with seven symmetrically-placed exit ports; six ports were connected via equal lengths of Tygon™ tubing to the respective inlets of the WBP chambers and the remaining port was used for sampling PM concentration. These components of the system were contained in an enclosed, vented Plexiglas^® ^box (4 ft × 4 ft × 4 ft) with the string generator, the six WBP chambers, and a real-time aerosol monitor (Figure 2).

The string generator has been shown to work most efficiently (i.e., high particle recovery) under low-moisture conditions [12]; therefore, a procedure for dehumidifying incoming compressed air was incorporated into this system. The air serving the generator was supplied by a medical grade, oil-less compressor (Powerex Oil-less Compressor OT50503; EdMac Compressor; Winston-Salem, NC). A dryer/filtration system (Del-Monox; Deltech; Ocala, FL) removed water from the airstream and purified the air of particles, organics, and CO_2_. Just prior to reaching the jets, air was deionized using a 2 mCi Po-210 aerosol neutralizer (Model P-2031-2000; NRD, LLC; Grand Island, NY) that served to cancel particle charge. The estimated final humidity of the air supplied to the system (<2%) served to eliminate: 1) particle clumping; and 2) excess humidification of the WBP chambers due to animal respiration. Temperature and humidity probes were inserted into one exposure and one control WBP chamber to permit characterization of the air conditions during exposure.

### Exposure configuration and validation

The aerosol generator operated at a flow rate of 13 L/min of air, with each of the six exposure WBP chambers receiving approximately 2 L/min; the remaining 1 L/min of air was routed to the sample port. The resistance in the exhaust flow line for each WBP chamber was adjusted daily so that the internal pressures within all chambers were equivalent and slightly negative with respect to ambient air. The two control WBP chambers (not shown) were also supplied with 2 L/min of air and kept at similar atmospheric conditions as the exposure chambers. A 47 mm Teflon filter was attached in-line just distal to the exhaust port of each air and exposure WBP chamber in order to evaluate the accumulated PM mass for each day. The aerosol concentration from the vertical mixing chamber was determined gravimetrically once every hour (for approximately five minutes with an air flow of 1 L/min) using a 47 mm Teflon filter attached to the sampling port; when aerosol concentration was not being assessed gravimetrically (≈55 min/hr), a real-time aerosol monitor (Dust Trak; TSI, Inc.; St. Paul, MN) was used to assess concentration and make adjustments to the string generator when the particle concentration deviated from the target exposure concentration.

### Exposure analyses and statistical methods

Chamber filters were weighed daily both pre- and post-exposure to determine the accumulated mass of PM. In addition, PM mass and concentration were calculated using filters attached to the sample port for a given period of time and at a known constant flow rate. A hierarchal one-way analysis of variance (ANOVA) was performed to test for differences in accumulated mass for individual WBP exposure chambers (SAS; Cary, NC).

### Particles

The PM used in this study (HP12) was combustion-derived from an oil-fired power plant and has been described previously [13]. After initial grinding, the HP12 particles had a mass median diameter (MMAD) of 3.76 μm and a geometric standard deviation (GSD) of 2.16. Particles were further ground prior to exposure and particle size was determined using a 7-stage cascade impactor (Intox Products; Albuquerque, NM); this resulted in a MMAD of 1.19–1.95 μm and a GSD of 2.66–3.49.

### HP12 chemistry analyses

Selected chamber filters (Table 7) from the 1- and 4-day 13 mg/m^3 ^exposures (Phases 3 and 4) were analyzed for HP12 chemical composition to permit comparisons among: 1) exposure chambers; 2) exposure days and phases; 3) bulk and resuspended samples; and 4) various chemical analyses. Chemical analyses of the HP12 samples included measurements of both solid samples and their liquid extracts to provide elemental speciation data. Solid samples were characterized for total elemental content, anion and cation content, and carbon fraction ratios. Deionized water and 1 M HCl extractions, followed by elemental analysis of the supernatants, provided estimates of easily bioavailable and totally bioavailable metal content, respectively. While this speciation scheme is a rough approximation of bioavailability for PM samples of complex composition, it has proven useful in characterizing inhalation toxicology endpoints for various source and ambient particulates [14,20,21,28]. Extractions using 1.3 M HNO_3 _were also performed to further examine elemental solubility. In all, four types of chemical analyses were conducted: XRF, CF, ICP-OES, and IC.

**Table 7:** HP12 filter collection information for ICP analysis

**Date Collected**	**Exposure Chamber**	**Extract Solution**
3 January 2005	1	deionized water
	2	1 M HCl
	5	1.3 M HNO_3_
5 January 2005	1	deionized water
	2	1 M HCl
	4	1 M HCl
	5	1.3 M HNO_3_
6 January 2005	2	deionized water
	3	1 M HCl
	4	deionized water
	5	1.3 M HNO_3_
7 January 2005	1	deionized water
	2	1 M HCl
	3	1 M HCl
	4	1.3 M HNO_3_
8 January 2005	1	deionized water
	3	1 M HCl
	4	1.3 M HNO_3_

### Total elemental analysis using XRF

Gelman Teflo filters (1 μm nominal pore size, 47 mm diameter; Pall Gelman Sciences; Ann Arbor, MI) containing deposited HP12 accrued during the 4-day 13 mg/m^3 ^exposure, along with five lot-matched blank filters, were analyzed using the Lawrence Berkeley Laboratory XRF Spectrometer (Lawrence Livermore National Laboratory; Livermore, CA). This analytical method follows the principles of EPA guidance [29] to quantify 47 elements.

### Analysis of carbon using CF

Carbon fractionation was used to separate the C content of the HP12 bulk samples into organic, elemental, and carbonate C. The thermo-optical method, based upon sequential pyrolytic vaporization and detection of the three carbon fractions [30,31], was performed by Sunset Laboratory (Forest Grove, OR).

### Analyses of extractions using ICP-OES and IC

Bulk and filter HP12 were extracted with 15 ml of either deionized water or 1 M HCl at 20°C and agitated using a 12-rpm end-over-end rotator (Model 4152110; Barnstead/Thermolyne; Dubuque, IA) for one hour. Extraction conditions using 1.3 M HNO_3 _were the same as those for deionized water/1 M HCl except for contact time duration (72 hours); the increase in agitation time was to allow for enhanced solubility. Bulk extractions were conducted using 80 mg of HP12, while filter extractions were performed on relatively small quantities of HP12 (range 246–652 μg). Both sample types of HP12 dispersed readily into all three extraction solutions, which resulted in evenly-mixed suspensions with no visible particle agglomeration. High-speed centrifugation (51000 × *g*) was used to separate the extraction supernatant from the solid portion. Liquid aliquots and dilutions were measured gravimetrically using audited balances and calibrated pipettors. For diluted deionized water extracts, the pH (Model 440; Corning Incorporated; Corning, NY) was acidic (4.2).

After dilution, the extraction supernatant was analyzed quantitatively for 31 elements using ICP-OES (Model P4300DV; Perkin Elmer Instruments; Shelton, CT) following EPA methodology [32]. Diluted deionized water extracts from the ICP-OES sample preparation were analyzed quantitatively for anion and cation content using IC. A DX-500 ion chromatograph (Dionex; Sunnyvale, CA) was used with an AS14 column (Dionex) for anion analysis and a CS12 column (Dionex) was used for cation analysis.

## Competing interests

The author(s) declare that they have no competing interests.

## Authors' contributions

LBW designed and coordinated the experiments, performed statistical analysis, and drafted the manuscript. ADL developed the exposure system and operated the whole-body plethysmography acquisition system. JKM and RBK performed the HP12 chemical analysis, interpreted the findings, and reported the results. WHR and JPN assisted in data collection and analysis. DLC and WPW conceived the study and contributed to the drafting of the manuscript. All authors read and approved the final manuscript.
